# Editorial: Channel Modulation in Neurodegeneration and Neuroprotection

**DOI:** 10.3389/fphar.2022.872103

**Published:** 2022-03-23

**Authors:** Jacques Joubert, Sarel F. Malan, Werner J. Geldenhuys

**Affiliations:** ^1^ Pharmaceutical Sciences, School of Pharmacy, University of the Western Cape, Bellville, South Africa; ^2^ Department of Pharmaceutical Science, School of Pharmacy, West Virginia University, Morgantown, WV, United States

**Keywords:** ion channels, neurological disorders, channelopathies, electrophysiology, neuropharmacology

Ion channels are critically important for the normal function of the brain and excitable tissues. The Research Topic “*Channel Modulation in Neurodegeneration and Neuroprotection*” focused on the role of ion channel function or dysfunction in neurological and neurodegenerative conditions. Convergent efforts to establish a link between clinical neurology, genetics, loss of function of important proteins and channelopathies in neurological disorders have become an intense area of research interest. Several ion channels have been implicated as important players in these diseases. This research topic therefore includes ten key research articles and two up-to-date review papers in the field of ion channels, their structural features and their proposed modes of action, through the analyses of their structural characteristics, structure-function relationship, therapeutic modulation and neuropharmacology.

In this collection, several authors focused on exploring and further elaborating on glutamate receptor channels and their role in the development and treatment of neurological disorders. Gale et al. investigated whether glutaminergic N-methyl-D-aspartate (NMDA) receptor channel antagonists, other than memantine, are able to treat patients with GRIN mutations on the GluN2A subunit of the NMDA receptor by attenuating neurotoxicity associated with GluN2A-P552R expression. The authors found that treatment with ketamine does not effectively block GluN2A-P552R-mediated dendrotoxicity, despite the fact that both memantine and ketamine act as open NMDA receptor channel blockers binding at the phencyclidine binding site. These findings suggest that GluN2A-P552R induced dendrotoxicity is mediated through two distinct mechanisms that are yet to be elucidated. The group of Sebih et al. synthesised the glutathione (GSH) metabolite gamma-L-glutamyl-L-glutamate (γ-Glu-Glu) and explored its effects on activation of NMDA receptors. They observed that γ-Glu-Glu partially activated NMDA receptors and exhibited better efficacy for NMDA receptors containing the GluN2B subunit. γ-Glu-Glu was also found to potentiate glutamate responses on NMDA receptors. Further experiments revealed that extracellular γ-Glu-Glu concentration was directly linked to GSH metabolism, suggesting that γ-Glu-Glu could exert excitatory effects when GSH production is enhanced, leading to the overactivation of neuronal NMDA receptors. The research article by Dron et al. described the effect of different anticonvulsants on native glutaminergic calcium-permeable α-amino-3-hydroxy-5-methyl-4-isoxazolepropionic acid receptor (AMPA) receptors (CP-AMPARs) and calcium-impermeable AMPA receptors (CI-AMPARs) using a whole cell patch-clamp method. Amongst the ten anticonvulsants evaluated, phenytoin was the only one with significant ability to reversibly inhibit CP-AMPARs and Cl-AMPARs. The authors have shown, for the first time, that AMPA receptor inhibition by phenytoin may contribute to its anticonvulsant ability, as well as to its side effect profile.

Dysregulation of Ca^2+^ and Na^+^ homeostasis has been described as being central in the etiology of neurodegenerative and neurological diseases. Much however remains unknown in the pathogenesis of these disorders and the role of ion exchangers, ion channels and ion release in the mechanism. In an paper by Piccialli et al. the authors demonstrate enhanced reverse-mode Na^+^-Ca^2+^ exchanger (NCX3) activity, dependent on interaction with the voltage gated sodium channel, Nav1.6, in primary hippocampal neurons of the Tg2576 mouse model for Alzheimer’s disease. Even though it is not entirely clear whether this is the result of a mutation associated with the Tg2576 mouse or a compensatory mechanism, the authors propose that the upregulation of NCX3 exerts a neuroprotective effect in amyloid beta (Aβ) induced calcium dyshomeostasis by enhancing the endoplasmic reticulum (ER) Ca^2+^ content and thus preventing ER-stress. Katnik and Cuevas, investigated whether inhibition of the sodium-potassium-chloride cotransporter-1 (NKCC1) by its antagonists, bumetanide and ethacrynic acid, would affect Na^+^ and Ca^2+^ overload following *in vitro* ischemia-acidosis. They concluded that it is the inhibition of voltage-gated Ca^2+^ and Na^+^ channels by these loop diuretics, and not the inhibition of NKCC1, that results in the reduction of [Ca^2+^]i overload in neurons during ischemia-acidosis. A review paper by Li et al. describes the post-translational modification of the Cav1.2 channel and its functions in neurodegenerative diseases. The authors expand on the potential of dihydropyridine Cav1.2 inhibitors that have recently been repurposed for the treatment of Parkinson’s- and Alzheimer’s disease.

Sodium and potassium channels are of significant interest in conditions such as neuropathic pain, neuroinflammation, migraine and epilepsy. Wang et al. report that the flavonoid Gastrodin, the bioactive ingredient of Gastrodia, a Chinese Herbal medicine used as an analgesic, effectively treats vincristine induced thermal and mechanical hyperalgesia in rats. These effects seem to be mediated by inhibition and/or suppression of expression of Nav1.7- and Nav1.8 channels in dorsal root ganglion neurons. Further *in silico* modeling showed possible direct binding of Gastrodin to the Nav1.7- and Nav1.8 channel in active sites previously associated with its inhibition. In the manuscript by Yao et al., the authors performed a genetic screen of 26 patients who suffer from febrile seizures through *de novo* sequencing and identified a novel missense mutation of the high-conductance calcium- and voltage-dependent K^+^ (BK) potassium channel gene KCNMA1 (E155Q). Electrophysiological characterization of different KCNMA1 mutants in HEK293T cells, the previously-reported R458T and E884K variants, and the newly-found E155Q variant, revealed characteristics of loss-of-function. Transcriptomic analysis on the hippocampus and cortex of BK knock-out and wild-type mice showed differentially expressed genes distributed in neuroinflammation, astrocyte activation, and epilepsy-related signal pathways. Eren-Koçak and Dalkara wrote a review on the possible contribution of ion channels to excitation-inhibition imbalance and neuroinflammation as a common mechanism for ion channel dysfunctions in migraine and depression. Finally, in an interesting opinion piece by Gascoigne et al. the authors are of the opinion that If chloride channels and/or GABAA receptors become dysfunctional, a lack of inhibitory control over neuronal activation is accompanied by a weakened hemodynamic response. Furthermore, they imply that such dysfunction is an important molecular hallmark of neurodegenerative and neurological disorders.

Two papers, published by Pesti et al. and Lukacs et al. describe unique approaches to identify the biophysical properties and detailed mechanism of action of voltage-gated sodium channel (VGSC) blockers using automated high-throughput screening compatible microfluidics-based patch clamp methods. The group showed that with their methods, they are able to distinguish state-dependent association, dissociation kinetics and identify concentration independent effects of VGSC blockers on time scales ranging from milliseconds to seconds. These two examples of automated patch clamp analysis may assist in the assessment of potential therapeutic agents for sodium channel induced hyperexcitability-related neurological disorders.

In summary, this Research Topic contributes to an “*in-depth*” knowledge of ion channels, their dysregulation and their effects in neurological conditions, opening the way to new attractive research in neuropharmacology, in parallel with the identification of new candidate drugs. We believe that this collection of articles will inspire many researchers and clinicians worldwide to continue working in or enter the field of ion channel research associated with neurological disorders.

